# Automated Detection of Actinic Keratoses in Clinical Photographs

**DOI:** 10.1371/journal.pone.0112447

**Published:** 2015-01-23

**Authors:** Samuel C. Hames, Sudipta Sinnya, Jean-Marie Tan, Conrad Morze, Azadeh Sahebian, H. Peter Soyer, Tarl W. Prow

**Affiliations:** Dermatology Research Center, School of Medicine, University of Queensland, Translational Research Institute, Princess Alexandra Hospital, Brisbane, Australia; Queen Mary Hospital, HONG KONG

## Abstract

**Background:**

Clinical diagnosis of actinic keratosis is known to have intra- and inter-observer variability, and there is currently no non-invasive and objective measure to diagnose these lesions.

**Objective:**

The aim of this pilot study was to determine if automatically detecting and circumscribing actinic keratoses in clinical photographs is feasible.

**Methods:**

Photographs of the face and dorsal forearms were acquired in 20 volunteers from two groups: the first with at least on actinic keratosis present on the face and each arm, the second with no actinic keratoses. The photographs were automatically analysed using colour space transforms and morphological features to detect erythema. The automated output was compared with a senior consultant dermatologist’s assessment of the photographs, including the intra-observer variability. Performance was assessed by the correlation between total lesions detected by automated method and dermatologist, and whether the individual lesions detected were in the same location as the dermatologist identified lesions. Additionally, the ability to limit false positives was assessed by automatic assessment of the photographs from the no actinic keratosis group in comparison to the high actinic keratosis group.

**Results:**

The correlation between the automatic and dermatologist counts was 0.62 on the face and 0.51 on the arms, compared to the dermatologist’s intra-observer variation of 0.83 and 0.93 for the same. Sensitivity of automatic detection was 39.5% on the face, 53.1% on the arms. Positive predictive values were 13.9% on the face and 39.8% on the arms. Significantly more lesions (p<0.0001) were detected in the high actinic keratosis group compared to the no actinic keratosis group.

**Conclusions:**

The proposed method was inferior to assessment by the dermatologist in terms of sensitivity and positive predictive value. However, this pilot study used only a single simple feature and was still able to achieve sensitivity of detection of 53.1% on the arms.This suggests that image analysis is a feasible avenue of investigation for overcoming variability in clinical assessment. Future studies should focus on more sophisticated features to improve sensitivity for actinic keratoses without erythema and limit false positives associated with the anatomical structures on the face.

## Introduction

Histopathology is the gold standard for diagnosis of actinic keratosis, however it is rarely warranted given the low risk of a single lesion progressing to squamous cell carcinoma; even in a high risk population progression rates were lower than 1% per year[1]. Assessment for patient care, epidemiological study and research into treatment depends on subjective clinical examination, a process that has been shown to have inter- and intra- observer variability[2–6]. Additionally, a number of treatments are available including lesion and field directed modes[7].

If interobserver variability in assessment is high it stands to reason that the same field could be treated using different modalities, the same participant could be evaluated with a different actinic keratosis burden or the same response to a new treatment could be assessed differently. Further, treatment variability could extend to some lesions being treated in a given area and lesions equally suspicious being left untreated in other areas. The best patient outcomes depend critically on accurate and repeatable assessment. Diagnostic standardisation is a means to this end. Automated image analysis has the potential to eliminate inter-observer variability and is a potential first step towards standardising the diagnosis and care of actinic keratosis.

A number of image analysis techniques have been proposed and validated for various problems in dermatology, but have typically focused on imaging of single lesions. Dermoscopic images of lesions have been analysed using a variety of methods to extract pigment networks[8,9], detect telangiectasia[10], segment lesion borders[11,12], and to diagnose melanoma[13,14]. Reflectance confocal microscopy optical sections have been analysed to detect the dermal-epidermal junction[15], estimate keratinocyte density[16] and diagnose melanocytic lesions[17].

Most promising for actinic keratosis assessment is the work of Cho et al.[18] and Lee et al.[19]. Both reported systems for counting nevi in clinical photographs of large body regions as opposed to single lesions in dermoscopic or closeup clinical images. Each used different approaches but both validated their process by comparison with dermatologist assessment; reported sensitivity and diagnostic accuracy were 84.7% and 79.3% for Cho et al. and 91% and 90% for Lee et al.

Since many of the signs of actinic keratosis are visual, including erythema, it was hypothesized that image analysis of clinical photographs could detect actinic keratoses similarly to an experienced dermatologist. In this pilot study a method for evaluating actinic keratosis using automated analysis of clinical photographs is proposed. The proposed algorithm is based on a colour space transform and morphological analysis to extract regions of skin displaying more erythema than surrounding skin. This approach was validated by directly comparing the automated output to the evaluation of the same images assessed by an experienced dermatologist, including the intra-observer variability of the dermatologist.

## Methods

### Volunteer recruitment

Two groups of volunteers were recruited: a high actinic keratosis group wherein each member had at least one actinic keratosis lesion on the face and each forearm, and a no actinic keratosis group with no clinically detected actinic keratoses. Volunteers were included in each group based on the number of actinic keratoses detected. Volunteers were not eligible to participate if they had been treated for actinic keratoses in the study areas within the previous three months at the time of photography. Each group had 10 members; the high actinic keratosis group consisted of 8 males and 2 females (mean age: 62.2, range 44–84 years), whereas the no actinic keratosis group consisted of 3 males and 7 females (mean age: 31.8, range 19–42 years). The study was approved by the Metro South and North Health Service Districts Human Research Ethics Committees (Approval: HREC/11/QPAH/236 and HREC/10/QPCH/181) according to the Declaration of Helsinki and informed consent was given in writing by the volunteers.

### Clinical photography

Clinical photographs were acquired using a digital SLR (550D, Canon, Tokyo, Japan) equipped with a telephoto lens (EF-S 60mm F/2.8 macro, Canon, Tokyo, Japan). Five photographs were acquired of each volunteer in standardized views: the front of the face, left and right profile views of the face, and the left and right arm, spanning from the tips of the fingers to past the elbow, with the elbow rotated so that the most sun exposed regions of the arm and hand were presented to the camera. The background was a standard white backdrop and lighting was provided by two studio flash lamps (Bowens Gemini 400s) each passing through a 0.8 m diameter octagonal soft box. The lights were positioned approximately 1 m from the subject to the left and right at 45 degrees to provide soft and even lighting of the skin with minimal reflections, glare and shadowing. The camera to subject distance was approximately 1.5 m, this varied slightly in order to appropriately frame the region of interest. The final image scale ranged from 9 to 11 pixels per millimetre. In total there were 100 photographs – 30 for each group of the face, and 20 of each group for the hands and arms.

### Dermatologist assessment

Each image was assessed by a senior consultant dermatologist (HPS), with the boundaries of actinic keratosis lesions digitally annotated on the original, full resolution image file. The dermatologist circumscribed distinct actinic keratosis lesions greater than 2 mm in size occurring on the arms and hands above the elbow, and on the face excluding the neck. 686 distinct actinic keratoses were identified in the high actinic keratosis group, with 557 (81%) occurring on the hands and arms. To incorporate the known variability in assessment of actinic keratosis, the images were labelled twice with at least two weeks in between sessions to minimize the bias introduced by memory of the previous labelling.

### Automated actinic keratosis location method

**Figure 1:**
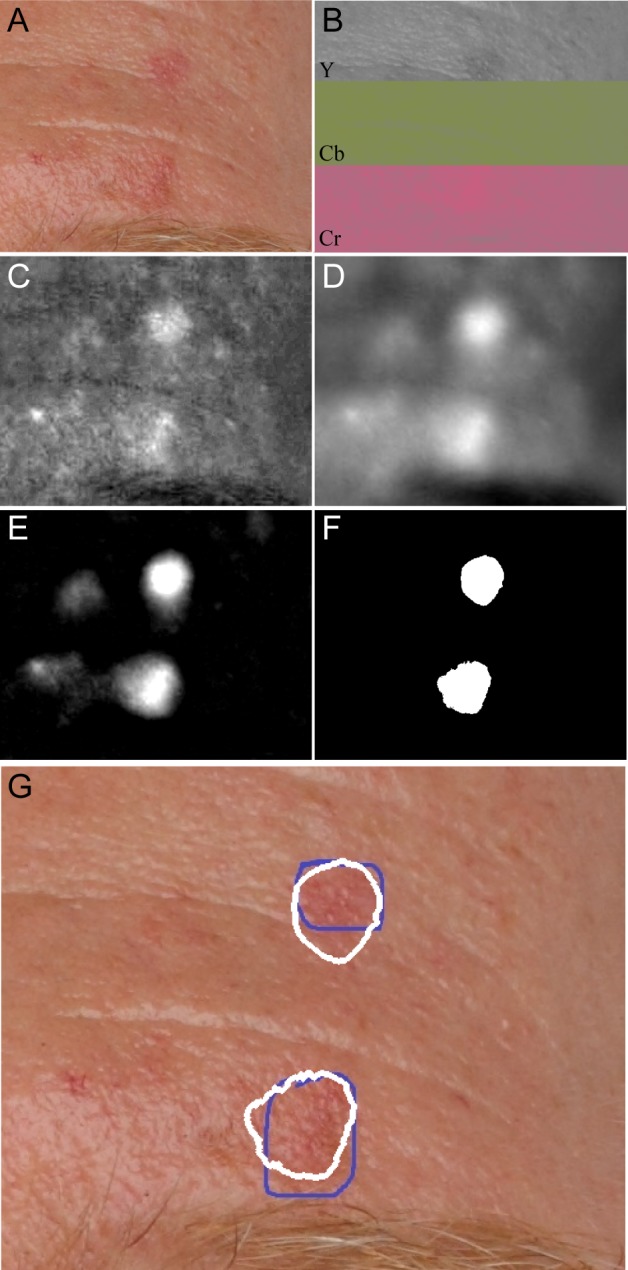
Overview of analysis steps in automated actinic keratosis detection, as applied to the dorsum of hand with the contrast adjusted for visualization. A) Input image. B) YCbCr transform of input image. C) Mean of Cb and Cr channels shows distinct hotspots for erythema. D) Guided filtering to remove unneeded texture. E) Distinct peaks extracted by morphological analysis. F) Hysteresis thresholding to identify erythematic areas. G) Boundaries of automatically detected lesions (white) compared with the dermatologist’s annotations (blue).

The automated method focused on identifying the erythema associated with actinic keratosis lesions, the main operations are visualized in **Fig. 1**. In detail, the YCbCr transform of the input RGB image was computed, and the mean of the Cb and Cr channels taken as the erythema intensity image. This image was smoothed using guided filtering[20] to remove unimportant textural variation. The peaks in this smoothed image were extracted by taking the difference of the smoothed erythema intensity image and the same image morphologically opened-by-reconstruction[21] with a disk. Hysteresis thresholding[22] was applied to the extracted peaks to discard low intensity regions while maintaining detected lesion shape. The output of this process was a binary image where true pixels indicate the regions likely to be actinic keratosis. All image processing was performed in Matlab (Version R2012b, The Mathworks Inc., Natick, Ma., USA). A reference implementation is provided in **S1 Compressed Archive.**

The final parameters for each component in the process were selected by a grid search with nested leave-one-out cross validation on the volunteers and using only the photographs in the high actinic keratosis group. The search examined guided filter square window sizes between 0 pixels (no smoothing) to 61 pixels and texture regularization parameter ϵ between 0.01 and 1. The radius of the disc for opening by reconstruction was varied between 10 and 70 pixels, and the low and high thresholds for hysteresis were varied between 0 and 0.008, and 0.001 and 0.012 respectively. The inner cross validation loop was used to select the parameters that maximized F2, the harmonic mean of sensitivity and positive predictive value (PPV), with sensitivity weighted twice as highly as PPV. Sensitivity and PPV were measured on a pixel basis with respect to the annotations made by the dermatologist. Sensitivity was calculated as the fraction of dermatologist labelled actinic keratosis pixels also labelled by the automated method, and PPV was calculated as the fraction of automated pixels that were also labelled by the dermatologist. The photographs of the face and arms were analysed separately.

### Comparison with dermatologist in the high actinic keratosis group

The automated method was compared to the dermatologist’s assessment by (1) whether the distinct lesions identified by each approach were co-local to each other (count measurement) and (2) how well the total lesions counted in each photograph by each method were correlated by Pearson’s correlation coefficient (correlation measurement). For the count measurement a lesion was defined as a connected group of pixels surrounded completely by non-lesional skin, and a lesion identified by the automated method was considered co-localized with a lesion identified by the dermatologist if the centroid of one lesion was contained within the border of the other or vice-versa. If more than one automatically detected lesion matched a single dermatologist identified lesion, only the first was counted as a match and the remainder were removed from consideration to avoid double counting matches. The correlation measurement was calculated as the Pearson correlation coefficient between the automated method and the dermatologist.

For the count measurement, performance was calculated as sensitivity (fraction of dermatologist identified lesions co-localized by the automated method) and PPV (fraction of automatically identified lesions co-localized by the dermatologist), both of these measures were calculated using the leave-one-out process determined parameters to limit over-fitting giving an optimistic bias to the results. To incorporate human variability as a reference, the sensitivity and PPV were also calculated between the two sets of dermatologist annotations, using identical definitions and measurements as for the automatic/dermatologist comparison. The correlation between the two dermatologist annotations was computed in the same way as for the automated/dermatologist comparison.

### Comparison of high and no actinic keratosis groups

Finally, the automated output on the severe and low photodamage groups was compared using a two sided t-test for the difference of the means, considering both the area and lesions counted per image. Comparing these two groups provides an indication of how well the automated method supresses false positives for the extreme case of no lesions being present.

### Statistical Analyses

The agreement between the automated method and the dermatologist and the repeat reliability of the dermatologist were assessed by calculating Pearson’s product moment correlation coefficient between the two groups. Significance was assessed by determining the probability that the observed slope was significantly different from zero (no correlation between the two methods). The difference between the high actinic keratosis and no actinic keratosis groups was assessed using Welch’s t test to account for the differing standard deviations of the high and no actinic keratosis groups. Statistical calculations were performed in Prism (Version 6, GraphPad Software, La Jolla, Ca., USA).

## Results

### Parameter estimation

**Figure 2:**
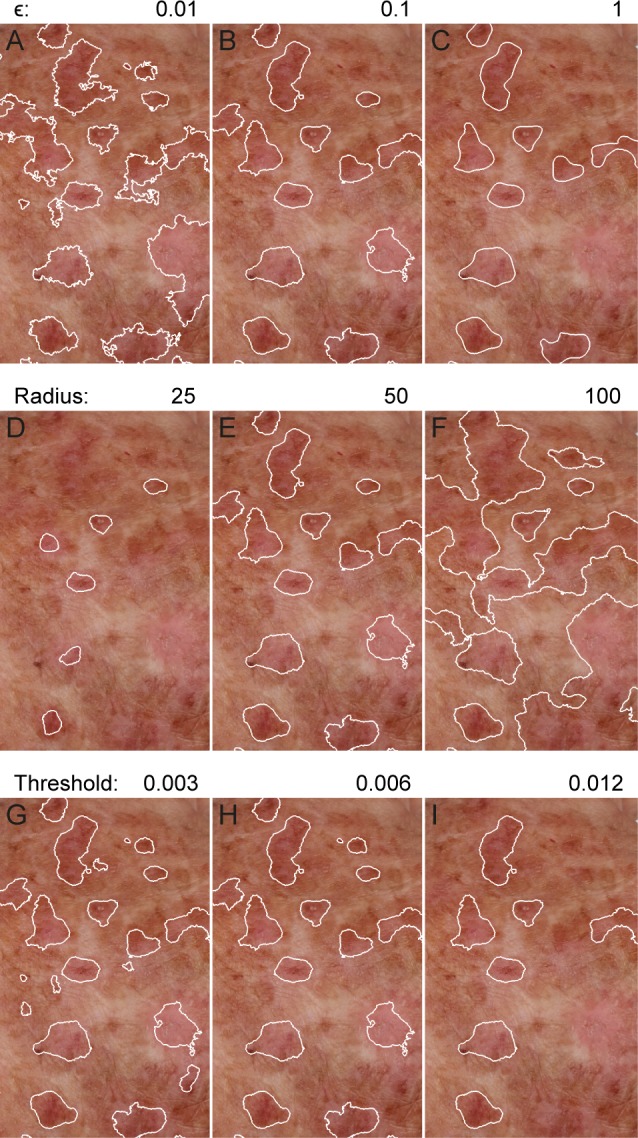
Impact of different parameters on automatically identified actinic keratosis lesions. A-C) The texture regularization parameter ϵ controls how smooth the detected lesions boundaries are. D-F) The radius of the disc used in morphological opening by reconstruction controls the size of detected lesions. G-I) The high hysteresis threshold controls whether or not a potential lesion is included based on the maximum erythema intensity.

The impacts of varying the guided filter regularization ϵ, the radius of the disc used to extract peaks, and the high hysteresis threshold are illustrated in **Fig. 2.** The grid search optimization process yielded different parameters for optimal detection in the face and arm groups. For the face, optimal parameters were: a guided filter size of 41 pixels, regularization ϵ of 1, disc size for reconstruction of 60 pixels radius and high and low hysteresis threshold of 0.007 and 0.005 while for the arms the parameters were: a guided filter size of 21 pixels, regularization ϵ of 0.1, disc size for reconstruction of 50 pixels radius and high and low hysteresis threshold of 0.008 and 0.003.

### Comparison with dermatologist in the high actinic keratosis group

On the photographs of the face, comparing the automated method with the dermatologist gave leave-one-out sensitivity and positive predictive value of 39.5% and 13.9% respectively. By comparison, the two dermatologist annotations gave sensitivity and positive predictive value of 65.9% and 54.5% respectively. For the photographs of the arm, the automated method had sensitivity and positive predictive value of 53.1% and 39.8%, with corresponding dermatologist results of 75.9% and 66.6%. The complete confusion matrices are shown in **Table 1**. The per image lesion counts for all methods are provided in **S1 Table.**

**Table 1:** Confusion between automated/dermatologist assessment and the first and second dermatologist assessments on the face and arms.

			**Automated**		**Dermatologist Count 2**	
		**Number of lesions**	**Present**	**Absent**	**Present**	**Absent**
**Dermatologist Count 1**	**Faces**	**Present**	51	78	85	44
		**Absent**	316	n/a	71	n/a
	**Arms**	**Present**	296	261	423	134
		**Absent**	447	n/a	212	n/a

**Figure 3:**
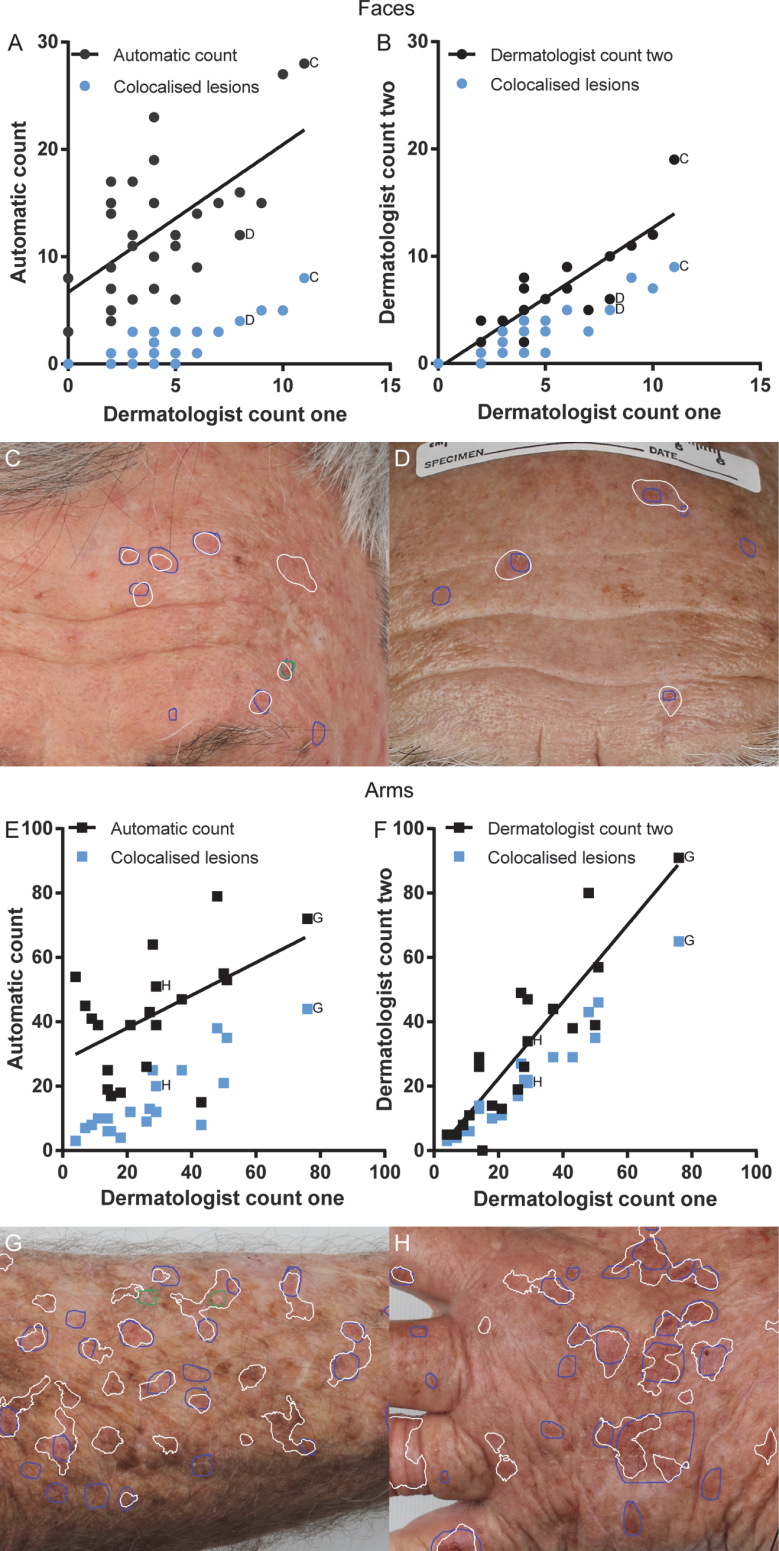
Comparison of automatically detected lesions with dermatologist circumscribed lesions for each body site. Each point represents the total count for each method from one clinical photograph in the severe photodamage group. Labelled points correspond to photographs shown. White outlines are automatically detected regions, blue/green outlines are dermatologist annotations. A) Automatically counted lesions on each face photograph compared with dermatologist count, and number of co-localized lesions. B) Dermatologist second count on faces compared with first count, and number of co-localized lesions. C-D) Example of automated output compared with dermatologist on two foreheads. E) Automatically counted lesions on each arm photograph compared with dermatologist count, and number of co-localized lesions. F) Dermatologist second count on arms compared with first count, and number of co-localized lesions. G-H) Example of automated output compared with dermatologist annotation on forearm and hand.

Comparing the correlations between the different sets of data, the correlation between the automatic method and the dermatologist was 0.62 (slope significantly different from zero, *p* = 0.0003*)* on the photographs of the face and 0.51 (*p* = 0.021*)* on the photographs of the arm. The corresponding dermatologist intra-observer correlations were 0.88 (*p* < 0.0001*)* for the face and 0.89 (*p* < 0.0001*)* for the arms. The distributions of the counts for each method, along with the number of co-localized lesions for each case are shown in **Fig. 3.** Unlike the dermatologist, there is a non-zero offset in the number of lesions identified, even in images with low number of actinic keratosis lesions counted by the dermatologist.

False positives were frequently associated with anatomical structures on the hands and face, especially the corner of the eyes, nostrils, mouth and ears. False negatives were associated with lesions that presented with scaling but no erythema. Additionally, very large lesions were not always detectable.

### Comparison of high and no actinic keratosis groups

**Figure 4:**
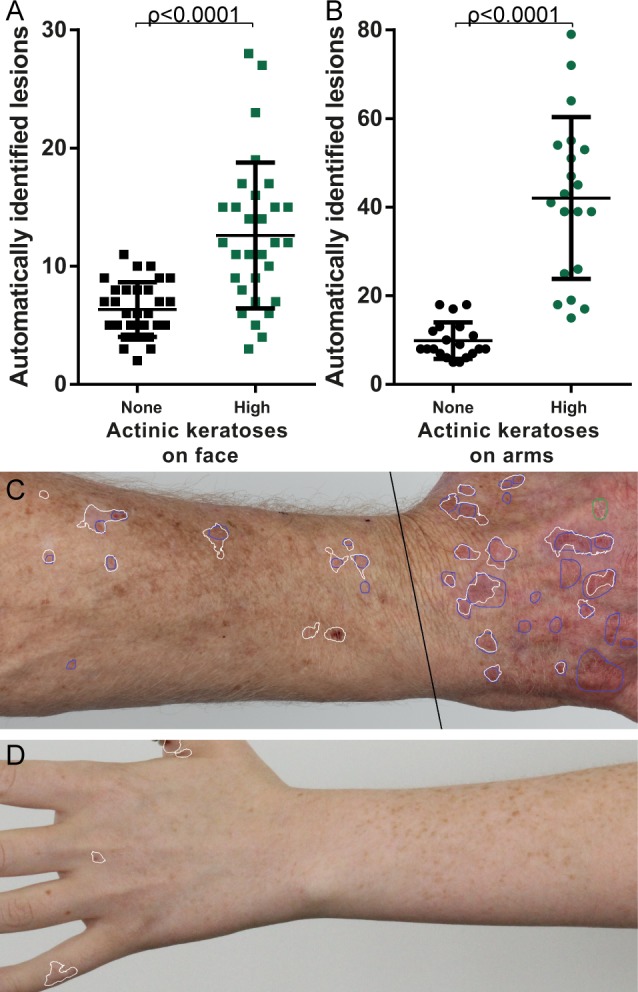
Comparison of automated detection output on low and severe photodamage groups, for all images. A) Lesions counted in each photograph of the face for both low and severe photodamage groups. B) Lesions counted in each photograph of the arm for both low and severe photodamage groups. C) Example output on hand and forearm from severe photodamage group. D) Example output on hand and forearm from low photodamage group.

Significantly more lesions were identified on the images of the face in the high actinic keratosis group (mean = 12.60, SD = 6.18) compared to the no actinic keratosis group (mean = 6.33, SD = 2.32); t(37.05)=5.199, *p* < 0.0001. This was also true for the images of the arm in the high actinic keratosis group (mean = 42.05, SD = 18.2) compared to the no actinic keratosis group (mean = 9.85, SD = 4.11); t(20.92) = 7.694, *p* < 0.0001. The counts over all images in each group are compared in **Fig. 4**. Although the means were significantly different for the faces there was still overlap between the groups, potentially due to the lower number of actinic keratoses present in the high actinic keratosis group (only 19% of the lesions were located on the face), and the higher number of false positives due to additional structure in the face.

## Discussion

The automated performance on the arms was better than on the face when comparing sensitivity (53.1% vs 39.5%) and positive predictive value (39.8% vs 13.9%). The very low positive predictive value on the face (13.9%) indicates that for every true positive there were 6.2 false positives or a severe over counting of the number of lesions present.

The assessment by the dermatologist also showed high variability of assessment over time. While the sensitivity and positive predictive values were higher than the automated method, there were still a large number of lesions identified on the second assessment that were not identified during the first assessment. Specifically on the arms 212 (33.4%) lesions were not identified during the first assessment, and on the faces 71 (44.5%) lesions were not identified during the first assessment.

This level of disagreement is higher than suggested by previous work assessing variability in terms of the agreement in the total number of lesions counted, rather than examining whether lesions identified were actually in the same location. Using the correlation coefficients to compare with other studies, the intra-observer agreement reported here (0.88 for the face and 0.89 for the arms) is directly comparable to Ianhez et al.[3] (0.93 for the face, 0.83 for the arms), and Atkins et al.[5] (0.7 on the arms, when counting lesions >5mm). Reported inter-observer agreements have been lower: Ianhez et al.[3] found agreement of 0.74 for the face, 0.77 for the arms, Chen et al.[4] found a total count correlation of 0.66 on the face and ears, and Lee et al.[2] found that after 4 years of consensus meetings the maximum agreement had reached 0.75 on the face and ears. By comparison, the correlation between automated assessment and dermatologist assessment was lower than all reported figures at 0.62 and 0.51 for the face and arms respectively.

Lee et al.[19] and Cho et al.[18] both developed systems for locating nevi in clinical photographs. Like in the approach reported here Lee et al. used no background detection or hair removal, while Cho et al. included sophisticated filtering for both. Lee et al. used a non-linear smoothing operation similar to the guided filtering used here, while Cho et al. selected potential nevi locations using a multi-scale difference of Gaussian detector. To select final lesions Lee et al. used heuristic filtering based on contrast, size and shape of pixel clusters; Cho et al. used a steerable pyramid filter description in the L*A*B* colour space and a support vector machine classifier, whereas the approach reported here used a simpler hysteresis thresholding which simplifies the task of classification but may limit performance.

Both Lee et al. and Cho et al. compared their detectors to experienced dermatologists, but because the face is not a major nevi location compared to the back neither Lee et al. nor Cho et al. needed to consider false positives due to anatomical structure. Lee et al. reported sensitivity of 90% on 8 photographs of the back; Cho et al. reported 84.7% sensitivity on 28 photographs of the back and arms, including the performance of their hair and background filtering. While the sensitivity reported here is lower (53.1% on images of the arm and 39.8% on images of the face) this is more representative of the challenge of actinic keratosis detection in general compared with locating melanocytic nevi.

This report includes the same variability in clinical assessment that has already been noted, and furthermore these results depend on the validity of photographic rather than clinical assessment; however, using the annotations on the clinical photographs does allow consideration of co-localization that would not be possible with a clinical diagnosis only. There is also no non-invasive tool to validate the clinical or photographic diagnosis with the same confidence as histopathology—ultimately alternative imaging tools such as reflectance confocal microscopy or optical coherence tomography may provide an objective validation of clinical diagnosis of actinic keratosis.

This automated analysis approach is limited by only focusing on erythema which is present in a variety of other unrelated skin conditions. This study design controlled for this by only investigating this feature in the context of clinically defined actinic keratosis in high actinic keratosis and no actinic keratosis groups without any other known skin conditions present. It is expected that other conditions characterized by erythema would cause false positives or that this approach could be used to characterize those lesions too, e.g. psoriasis. There was also no attempt made to detect keratotic scale, however with erythema as the only indicator 39.5% of the dermatologist identified lesions on the face and 53.1% of lesions on the arm were correctly co-localized by the automated analysis. Additionally, the number of participants in this study was small. While the nested leave-one-out cross validation can limit over-fitting and non-generalizable results, it cannot be ignored that this small sample may not be representative of actinic keratosis presentation in practice.

In conclusion, this automated method is not a substitute for expert dermatologist’s assessment of the same images. However, given the focus on a single, simple, feature (erythema) the sensitivity of actinic keratosis detection is promising, as is the correlation between automatic and dermatologist counts of lesions. Future studies should focus on developing more sophisticated features, both for detecting lesions that do not present with erythema, and suppressing false positives due to anatomical structures, especially on the face. The true agreement amongst observers also bears further investigation by taking into account whether counted lesions are actually in the same location.

## Supporting Information

S1 Table Lesions counted per image, across all images in the dataset. (CSV)

S1 Compressed Archive Sample Matlab implementation of the actinic keratosis detection algorithm. (ZIP)
